# Assessing outcomes after thrombectomy with or without iliac vein stenting for young provoked DVT patients with iliac vein stenosis

**DOI:** 10.1186/s12959-023-00537-9

**Published:** 2023-09-15

**Authors:** Hongji Pu, Jumin Song, Zhijun He, Fuyin Wang, Jiateng Hu, Sheng Huang, Minyi Yin, Weimin Li, Xiaobing Liu, Xinwu Lu, Guang Liu

**Affiliations:** 1 Department of Vascular Surgery, Shanghai Ninth People’s Hospital, Shanghai Jiao Tong University School of Medicine, No. 639 Zhizaoju Rd, Shanghai, 200011 P. R. China; 2 The Vascular Center, Shanghai Jiao Tong University School of Medicine, Shanghai, P. R. China; 3 Department of Vascular Surgery, Fengcheng Hospital, Shanghai Ninth People’s Hospital, Shanghai Jiao Tong University School of Medicine, Shanghai, P. R. China; 4 Department of General Surgery, Shanghai University of Medicine & Health Sciences affiliated Zhoupu Hospital, Shanghai, P. R. China; 5 Department of Orthopedics, Putuo Hospital of Zhejiang, Sir Run Run Shaw Hospital, Zhejiang University School of Medicine, Zhoushan, P. R. China

**Keywords:** Deep venous thrombosis, Iliac vein stenosis, Thrombectomy, Venous stents

## Abstract

**Background:**

This study aimed to assess the outcomes of thrombectomy with/without iliac vein stenting for young and transiently provoked DVT patients with iliac vein stenosis.

**Methods:**

This is a retrospective analysis of a prospectively collected multicenter database. Acute, transiently provoked DVT patients between 18 and 45 years old with iliac vein stenosis were included. All patients underwent thrombectomy. Outcomes including the Villalta score, the VEINES-QOL score, and adverse events were evaluated.

**Results:**

The data of 522 patients were collected of whom 75 were included, 58 underwent thrombectomy alone (nonstenting group) and 17 underwent thrombectomy and stenting (stenting group). Within 6 months, the Villalta score of patients in stenting group is lower than that of patients in nonstenting group (6 mo: 0.73 ± 0.77 vs. 1.41 ± 0.56, p = .0004), and the VEINES-QOL score of stenting group is higher than that of nonstenting group (6 mo: 89.00 ± 2.94 vs. 87.47 ± 3.72, p = .2141). At the following follow-ups, the Villalta score (12 mo: 0.56 ± 0.49 vs. 0.60 ± 0.58, p = .8266) and VEINES-QOL score (12 mo: 88.36 ± 2.29 vs. 88.31 ± 3.36, p = .9604) between the two groups are similar.

**Conclusion:**

The stenting group had better efficacy within 6 months after intervention, while there was no significant difference in the symptom, signs, and quality of life between two groups after 6 months within a 2-year follow-up.

**Trial registration:**

This study was registered in the Chinese Clinical Trial Registry (Registration Number: ChiCTR2200056073).

**Supplementary Information:**

The online version contains supplementary material available at 10.1186/s12959-023-00537-9.

## Introduction

Deep venous thrombosis (DVT) is the abnormal coagulation of blood in the deep venous system, with an annual incidence of approximately 1–2‰ [1–3]. Approximately 50% of DVT patients gradually develop a series of signs and symptoms called postthromobtic syndrome (PTS), which may reduce the quality of life of patients severely [1].

Venous thrombosis in the lower extremities may be associated with iliac vein stenosis. Previous studies noted that more than 75% of patients suffering from left lower extremity DVTs have iliac stenosis, such as iliac vein compression syndrome [4, 5]. The treatment strategy for acute DVT patients with iliac vein stenosis remains controversial, since interventional therapies appear to be no more effective than anticoagulant therapy alone [6, 7]. Stent implantation may be effective for acute DVT patients with iliac vein occlusion or stenosis greater than 50% after thrombectomy [8, 9]. However, the average age in existing studies were mostly over 50 years [5, 10, 11], and there have been fewer studies on younger patients [12]. Stenting is proved important in preventing venous fibrotic stenosis and maintaining venous outflow tract patency [8, 13], but evidence for stenting was lacking in young and acute DVT patients with sufficient venous return function.

In this study, we investigated whether stenting can be avoided in young and transiently provoked DVT patients with iliac stenosis more than 50% and good venous return (defined as the contrast agent stasis time of fewer than 6 s). The symptom remission, quality of life of the patients, and incidences of thrombosis and stent occlusion were compared between stenting and nonstenting groups. The results might provide data for the development of interventional strategies for young patients with provoked DVT.

## Methods

This study is reported with guidance of the Strengthening the Reporting of Observational Studies in Epidemiology (STROBE) statement [14].

### Study design and patient population

The data was prospectively collected from four medical alliance hospitals (Shanghai Ninth People’s Hospital, Fengcheng Hospital, Zhoupu Hospital, and Putuo Hospital). between January 2016 and December 2021. Acute, transiently provoked DVT patients between 18 and 45 years old with iliac stenosis who underwent AngioJet rheolytic thrombectomy (ART) and met the criteria were included. This study was approved by the Ethics Committee of Shanghai JiaoTong University School of Medicine.

The inclusion criteria were as follows: [1] age 18–45 years; [2] acute-phase DVT (within 2 weeks of symptoms onset) with iliac vein stenosis and received ART; [3] a clear provoking factor of DVT; [4] iliac-femoral DVT diagnosed for the first time, with or without femoropopliteal DVT and pulmonary embolism, and no history of superficial varicose veins or leg swelling. The exclusion criteria were as follows: [1] congenital diseases, such as Klippel-Trenaunay syndrome; [2] contraindications to anticoagulation or abnormal coagulation function; [3] incomplete treatment or missing data, and [4] iliac vein stenosis less than 50%, or contrast agent stasis time more than 6 s after ART and possible balloon dilation or catheter-directed thrombolysis (CDT).

### Procedures

The ART procedures have been described previously [5]. Briefly, patients received 200 U/kg low-molecular-weight heparin or 1.0 mg/kg enoxaparin following diagnosis. During the procedure, after the guidewire and the AngioJet catheter (Solent/Zelante, Boston Scientific, USA) were successfully advanced through the thrombosed vein segment, a power pulse lytic model was used with 0.25 × 10^6^ units of urokinase in 100 mL of saline. Fifteen minutes later, the AngioJet catheter was placed in standard rheolytic thrombectomy mode. This sequence was repeated if significant residual thrombi remained on subsequent venograms.

Some patients underwent balloon dilation and CDT after ART. The balloon dilation was performed using balloon catheters (Mustang, Boston Scientific, USA) with a diameter of 8–16 mm for serial dilation from the femoral vein to the common iliac vein. The procedure of CDT was inserting a multiple-side-hole infusion catheter (Angiodynamics, Queensbury, USA) into the thrombotic segment, and injecting urokinase (30,000–50,000 U/100 mL/h). When the fibrinogen level reached 1.5 g/L, the urokinase dosage was halved, and CDT would be finished when the fibrinogen level was decreased to 1.0 g/L. After ART and possible balloon dilation or CDT, venogram was performed to exam the iliac vein stenosis rate and contrast agent stasis time. The representative DSA sequence of contrast agent stasis time examination is shown in Supplemental file 1. The contrast agent stasis time is defined as the interval from the arrival of contrast agent in the iliac vein to the disappearance of contrast at the same level.

As described before [15], for patients underwent stenting, self-expanding stents Wallstent (Boston Scientific, USA) or a Lifestar (Bard Medical, USA) were implanted after balloon dilation. The stent diameters ranged from 12 to 14 mm for the iliac-femoral vein and from 14 to 16 mm for the common iliac.

After the procedure, patients of both groups received the same anti-coagulation therapy. Low molecular weight heparin was used during hospitalization. After discharge, warfarin or rivaroxaban were prescribed for at least 6 months since patients underwent endovascular intervention according to CIRSE standards of practice guidelines [8]. For patients taking warfarin, the international normalized ratio was maintained between 2 and 3. Patients received no postoperative anti-platelet treatment. All the patients used compression stockings (class II, 30 mmHg) as a standard adjunct treatment.

### Follow-up and outcomes

The outcomes included severity of lower limb symptoms, quality of life, free from re-thrombosis rate and complications. The severity of lower limb symptoms was evaluated using the Villalta score. The VEINES-QOL questionnaire was translated into local language from the appendix of Kahn 2006 [16]. Clinical follow-up visits were scheduled at 1, 3, 6, and 12 months after the procedure, and then yearly. A duplex scan or venography was performed to assess patency, and questionnaire follow-up was used to record the Villalta score and the VEINES-QOL score. Adverse events were continuously monitored during follow-up.

### Statistical analysis

Binary data are expressed as absolute or relative frequencies and were compared by the chi-square or Fisher exact tests. Ranked data are expressed as absolute values and were compared by the Cochran-Armitage trend test. Continuous data are expressed as the mean ± standard deviation and were compared using Student’s t-test. The free from re-thrombosis rate was assessed by the Kaplan–Meier estimator. For all tests, p < .05 was defined as significant. All analysis was performed using SAS 9.2 (Stat, SAS Institute, Cary, NC, USA).

## Results

### Patient demographics and procedure characteristics

The prospective database contained 522 patients, of whom 75 (14.37%) patients met the criteria and involved in the analysis. All involved patients were admitted to hospital and underwent the procedure during the acute phase of DVT. The baseline characteristics of the patients are reported in Table 1. The average age of patients who underwent stenting was 36.65 ± 4.61 years (range: 25–45) and that of patients without stenting was 32.52 ± 8.77 years (range: 19–45). All patients had transient provoking factors for DVT, including surgery, trauma, immobilization (≥ 72 h), pregnancy/puerperium and hormone therapy (such as oral contraceptive). In both groups, most of the thrombi occurred in the left extremity. A total of 58.82% of patients in the stenting group and 25.86% of patients in the nonstenting group underwent CDT (p = .0183). Except for the rate of CDT use, no other significant differences were observed in the baseline characteristics between the stenting and nonstenting groups. No vessel perforation, hemorrhage, severe pain, or rapid heart rate or blood pressure drop occurred during the procedure. All patients received therapeutic oral anticoagulation for at least 6 months after discharge, and there was no significant difference in the use of anticoagulants (p = .8268). Three patients in the stenting group and twelve patients in the nonstenting group requested switch from warfarin to rivaroxaban for ease of administration, and no adverse event was reported before and during the drug switch. No patient received anti-platelet therapy.

**Table 1:** The demographics and procedure characteristics of involved patients

Variables		Stenting (n = 17)	Nonstenting (n = 58)	p value
Age, y		36.65 ± 4.61	32.52 ± 8.77	0.0695
Sex (male)		82.35% (14)	63.79% (37)	0.2370
Provoking factor *				
	Surgery	17.65% (3)	17.24% (10)	0.5369
	Trauma	47.06% (8)	31.03% (18)	
	Immobilization	29.41% (5)	29.31% (17)	
	Pregnancy/puerperium	5.88% (1)	13.79% (8)	
	Hormone therapy	0 (0)	8.65% (5)	
Thrombus location**				
	Left	94.12% (16)	81.03% (47)	0.1957
	Right	5.88% (1)	18.97% (11)	
ART time, s		292.35 ± 88.10	304.76 ± 84.07	0.6032
CDT		58.82% (10)	25.86% (15)	0.0183
Postoperative anticoagulants				
	Rivaroxaban	64.70% (11)	67.24% (39)	0.8268
	Warfarin	17.65% (3)	12.07% (7)	
	Warfarin to Rivaroxaban ***	17.65% (3)	20.69% (12)	
Coagulation function during rivaroxaban				
	PT, s	13.22 ± 1.81	12.78 ± 1.54	0.3681
	APTT, s	33.43 ± 5.01	31.40 ± 5.84	0.2457
Coagulation function during warfarin				
	INR	2.42 ± 0.19	2.36 ± 0.25	0.7551
Follow-up time		15.06 ± 8.91	18.29 ± 9.44	0.2185

The data were expressed as mean ± standard deviation or percentage (number). ART: AngioJet rheolytic thrombectomy; CDT: catheter-directed thrombolysis

* “Trauma” included all patients had trauma and patients underwent surgery or had prolonged immobilization were excluded. Two patients had both immobilization and surgery, one patient had both immobilization and trauma, and the three patients were counted in “Immobilization”

** In the case of bilateral DVT, the leg with the most proximal localization was considered to be the index leg

*** Fifteen patients requested switch from warfarin to rivaroxaban within 4 weeks after discharge. No adverse event was reported during the drug switch

DSA images. (A) DSA image showed the presence of iliofemoral vein thrombosis before intervention. (B-C) Intraoperative imaging of ART and balloon dilation of iliofemoral veins. (D) After intervention, the proximal iliac vein was still stenosed. (E-F) Six-month follow-up angiography showed the proximal iliac vein occlusion and well-developed collateral vessels. DSA: digital subtraction angiography; DVT: deep vein thrombosis.

**Fig. 1:**
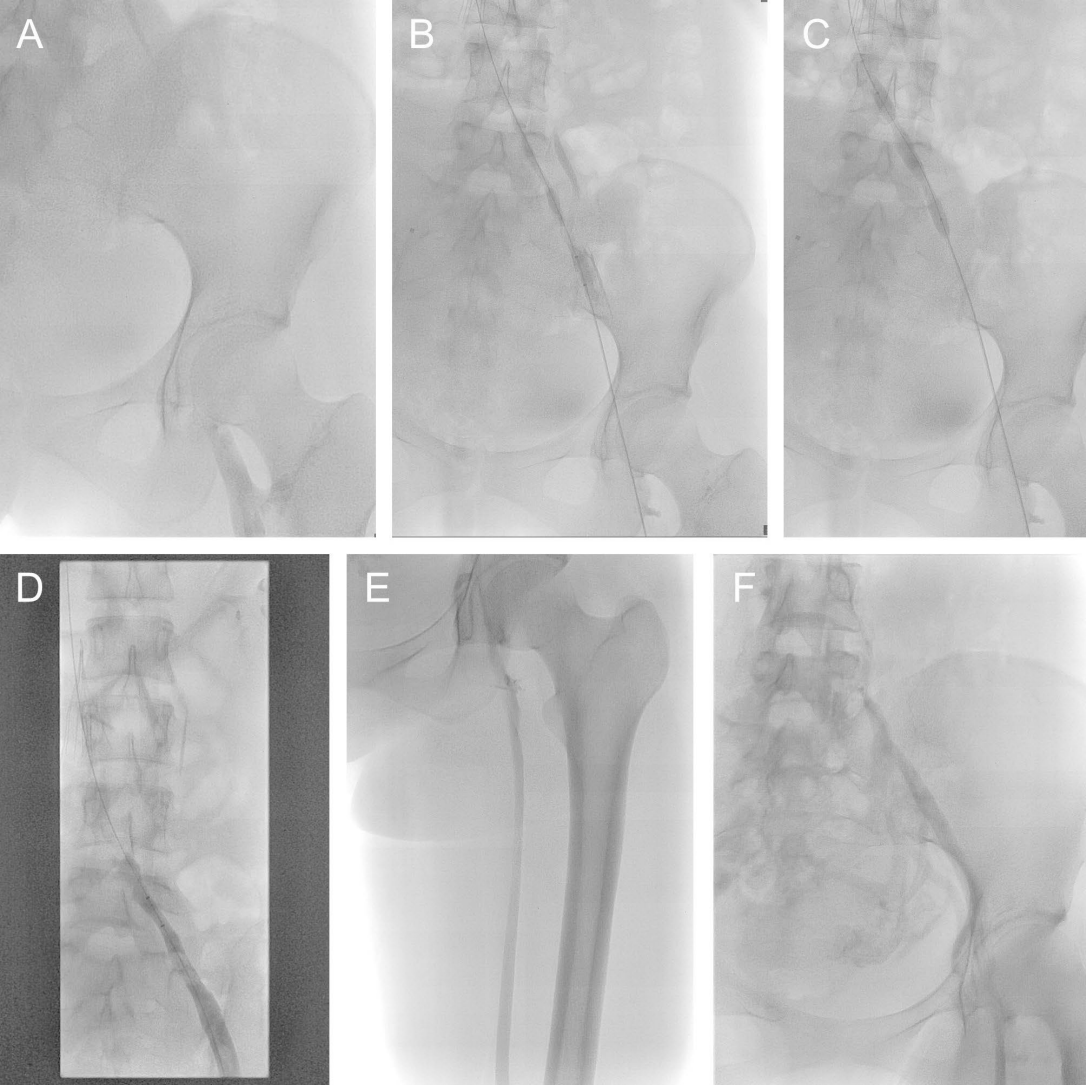
Representative DSA images of a patient underwent ART without stent. DSA images. (**A**) DSA image showed the presence of iliofemoral vein thrombosis before intervention. (**B-C**) Intraoperative imaging of ART and balloon dilation of iliofemoral veins. (**D**) After intervention, the proximal iliac vein was still stenosed. (**E-F**) Six-month follow-up angiography showed the proximal iliac vein occlusion and well-developed collateral vessels. DSA: digital subtraction angiography; DVT: deep vein thrombosis

The representative digital subtraction angiography (DSA) images of a patient underwent ART without stent are presented. The patient was a 23-years-old female, diagnosed as DVT provoked by pregnancy (Fig. 1A). ART and balloon dilation were performed as described above (Fig. 1B-C). After intervention, the proximal iliac vein stenosis was still more than 50% and the stasis time of the contrast agent in the left iliac vein was 5 s (Fig. 1D). Iliac vein stent was not implanted. Six-month follow-up DSA showed that well-developed collateral vessels were visualized while the proximal iliac vein stenosis presented (Fig. 1E-F). The patient had no symptoms or signs of heaviness, cramps, edema, and varicose veins.

### Outcomes

The Villalta score and VEINES-QOL score of young DVT patients with iliac stenosis received stent implantation or not after thrombus removal before intervention and during follow-up.

**Fig. 2:**
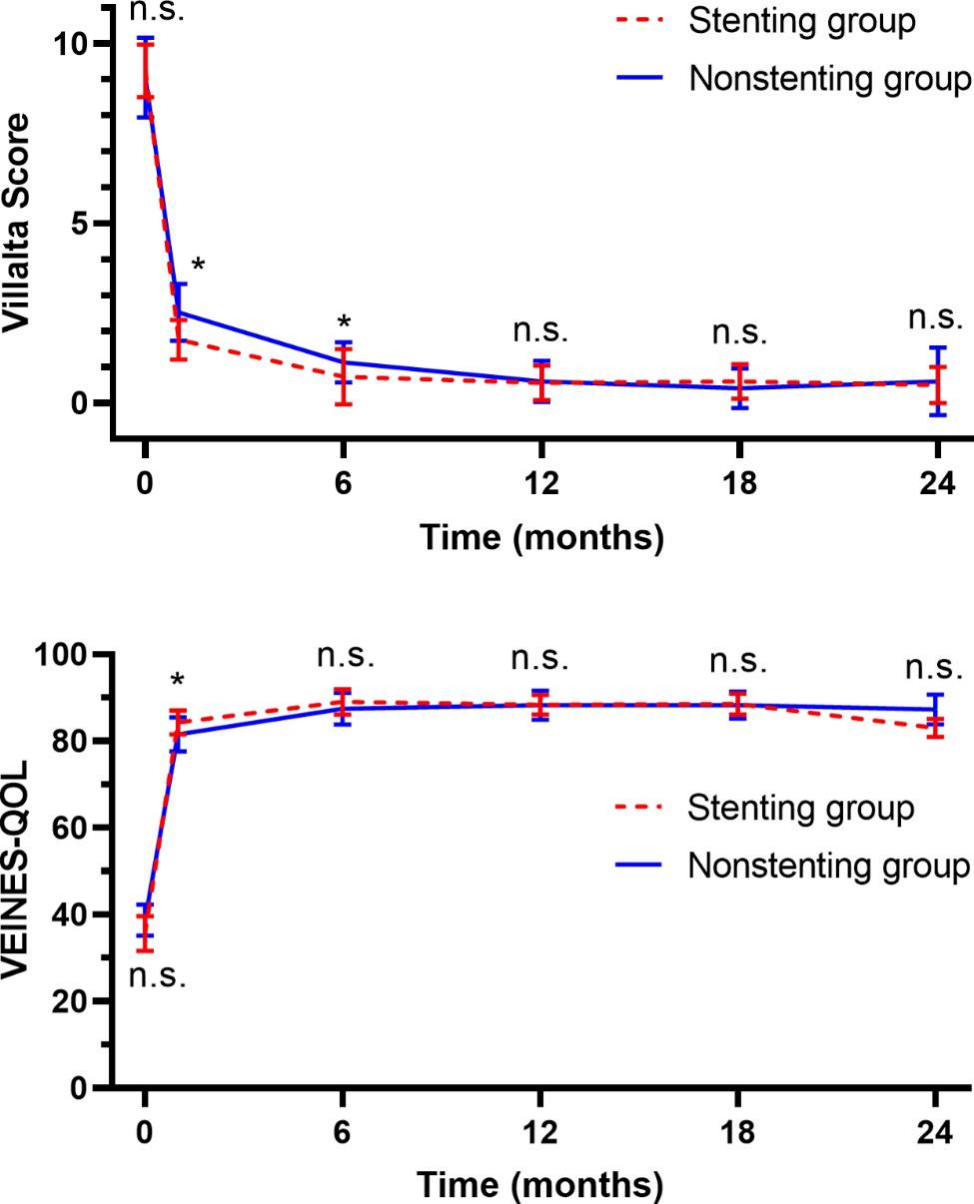
The clinical outcomes of patients. The Villalta score and VEINES-QOL score of young DVT patients with iliac stenosis received stent implantation or not after thrombus removal before intervention and during follow-up

The preoperative and follow-up outcomes are summarized in Table 2; Fig. 2. The Villalta score was used to quantify the severity of symptoms and signs. Before intervention, the Villalta scores of the stenting and nonstenting groups were similar (p = .5266). After intervention, the Villalta scores of both groups decreased. The Villalta scores of patients with stenting were significantly lower than those of patients without stenting at 1 month (p = .0005) and 6 months (p = .0004) after intervention. At month 12, the Villalta scores of the stenting group and nonstenting group had decreased further, and there was no significant difference between the two groups (p = .8266). At the 18- and 24-month follow-ups, the scores in both groups remained low, and there was also no significant difference between the two groups. During the follow-up, no patient was diagnosed with PTS in either group. The VEINES-QOL questionnaires were used to assess patients’ quality of life. Before intervention, there was no significant difference in the VEINES-QOL scores between stenting group and nonstenting group (p = .0576). The VEINES-QOL score of the nonstenting group was significantly lower than that of the stenting group at 1 month after intervention (p = .0094), and there was no significant difference between the two groups at the subsequent 6- (p = .2141), 12- (p = .9604), 18- (p = .8101) and 24- (0.0571) month follow-ups.

**Table 2:** The clinical outcomes of patients

Variables		Stenting (n = 17)	Nonstenting (n = 58)	p value
Before intervention				
	Villalta Score	9.24 ± 0.73	9.05 ± 1.11	0.5266
	VEINES-QOL	35.59 ± 4.00	38.67 ± 3.58	0.0576
1 month				
	follow-up rate	100.00% (17)	100.00% (58)	
	Villalta Score	1.76 ± 0.55	2.52 ± 0.79	0.0005
	VEINES-QOL	84.29 ± 2.78	81.53 ± 3.93	0.0094
6 months				
	Follow-up rate	94.12% (16)	93.10%% (54)	
	Villalta Score	0.73 ± 0.77	1.41 ± 0.56	0.0004
	VEINES-QOL	89.00 ± 2.94	87.47 ± 3.72	0.2141
12 months				
	follow-up rate	82.35% (14)	94.83% (55)	
	Villalta Score	0.56 ± 0.49	0.60 ± 0.58	0.8266
	VEINES-QOL	88.36 ± 2.29	88.31 ± 3.36	0.9604
18 months				
	follow-up rate	41.18% (7)	65.52% (38)	
	Villalta Score	0.60 ± 0.49	0.41 ± 0.55	0.4840
	VEINES-QOL	88.57 ± 2.44	88.26 ± 3.13	0.8101
24 months				
	follow-up rate	17.65% (3)	37.93% (22)	
	Villalta Score	0.50 ± 0.50	0.61 ± 0.95	0.8790
	VEINES-QOL	83.00 ± 2.16	87.27 ± 3.45	0.0571

The clinical outcomes of young DVT patients with iliac stenosis received stent implantation or not after thrombus removal

The Kaplan-Meier curves represent the occurrence of recurrent DVT within the follow-up duration. CI: confidence interval.

**Fig. 3:**
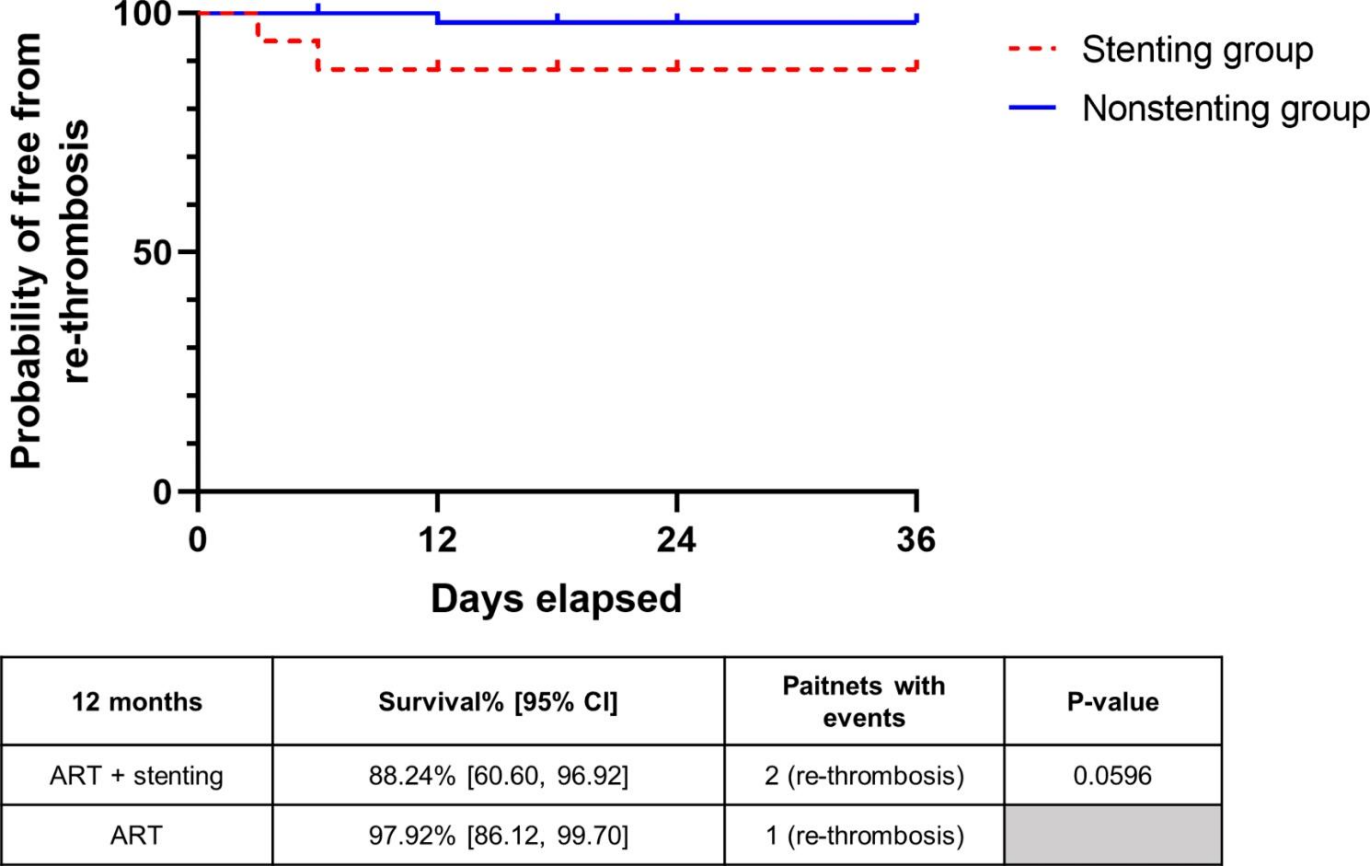
Probability of free from recurrent DVT or stent occlusion. The Kaplan-Meier curves represent the occurrence of recurrent DVT within the follow-up duration. CI: confidence interval

The proportion of patients free from recurrent DVT was represented by Kaplan–Meier survival curves (Fig. 3). For the stenting group, stent occlusion was also included among the events. According to the Kaplan–Meier curves, there was no significant difference in the rate of or recurrent DVT between the nonstenting group and the stenting group after 12 months (p = .0596). In the stenting group, 1 case of thrombosis was diagnosed by the 3-month follow-up, and another case was reported 6 months after intervention. The rethrombosis-free rate was 88.24% after 12 months. In the nonstenting group, one rethrombosis was reported in the 12 months after intervention, and the rethrombosis-free rate was 97.92%. No serious adverse events (defined as vessel perforation, iatrogenic embolization, other hospitalizations or death) occurred after the intervention.

## Discussion

Existing intervention strategies inadequately target the young secondary DVT population with iliac vein stenosis due to the low morbidity, small number of studies, and low sample size [17]. Although venous stenting has become a more commonly used treatment for iliac vein obstruction [18], the evidence supporting the use of stenting to prevent PTS and rethrombosis is insufficient. More targeted stent implantation was also conducive to reducing medical costs and improving the medical experience of young patients. In the real world, a considerable number of young people were reluctant to undergo stent implantation for the risk of rethrombosis, stent fatigue and instent stenosis. To meet the needs, more detailed strategy was supposed to be refined.

This study was conducted specially for young patients under 45 years old. Within 6 months after endovascular treatment, the degree of improvement in limb symptoms and quality of life in patients who underwent stenting was higher than that in patients who did not. At the 12-month and later follow-ups, there was no significant difference in clinical outcomes between the stenting and nonstenting groups. This result suggested that nonstenting was feasible for young patients with transiently provoked DVT, in cases of iliac vein stenosis more than 50% with proper collateral circulation after thrombus removal.

In patients with DVT provoked by a major transient risk factor, the risk of recurrent venous thromboembolism was low [19, 20]. The patients involved in this study had no previous symptoms of chronic venous insufficiency, and their DVT was triggered by obvious causes. In addition, the risk was also considered low in patients with an unprovoked isolated distal deep vein thrombosis [21]. For these patients, the venous return may recover if the thrombosis was cleared and the pelvic venous collateral circulation was re-established; 3–6 months of treatment after thrombosis was considered adequate [9, 19, 22]. In this study, the results indicated that within 6 months after the procedure, the Villalta score and quality of life of patients without stenting were lower than those of patients who underwent stent implantation. At subsequent follow-ups, the Villalta score and quality of life of the two groups converged and showed no significant difference.

Stenting may be beneficial and necessary for patients with severe iliac vein obstruction. In this study, only young DVT patients with clear provoking factor were included. For these patients, iliac vein stenosis may not be the direct factor provoking DVT. Therefore, it is notable that the result cannot be generalized to the whole population. For patients with DVT provoked by iliac vein stenosis, stent implantation might be the etiological treatment. Besides, only patients with good venous return underwent ART without stenting. Venous return function of iliac-femoral vein was assessed by the stasis time of the contrast agent whether exceeded 6 s during angiography. Six seconds was referred to the final stage of the femoral vein valvar reflux time phase in venous insufficiency, which was considered persistent reflux [23]. It was used for the first time as an index to evaluate the reflow efficiency of the iliac vein and collateral veins in DVT. Although the most appropriate time is not known, this is a relatively conservative attempt to minimize human-made thrombotic factors in clinical observation.

The limitations of the study should also be mentioned. The relatively small sample size resulted in wider confidence intervals regarding the estimates of the Villalta score and quality of life, and may obscure the differences between two groups. In other words, although current research indicates no significant difference between two groups after 6 months, potential differences may be revealed with the increase in sample size. Therefore, the findings should be interpreted with caution. Besides, during the procedure, we prescribed that the young patients had to undergo stenting when the stenosis of the femoral-iliac vein remained more than 50% and the stasis time of the contrast agent exceeded 6 s after CDT. However, the stricter and more detailed criteria were unknown, which required us to explore steadily and safely. Another limitation was the relatively short follow-up period of this study. The occurrence and development of PTS was a long-term process, and long-term follow-up was important for research on PTS. This study was ongoing, and future results may provide more evidence on this topic. Additionally, this study was subjected to uncontrolled confounding due to the heterogeneous nature of the cohort and the incomplete incorporation of variables, such as the lack of the grade of thrombus clearance. The presence of these confounding may potentially contribute to reduced confidence. Overall, this study was a pilot study, and further randomized controlled studies were needed to verify the conclusions and define more detailed treatment criteria.

## Conclusion

Young DVT patients with iliac vein stenosis needed to be treated with caution. Our study indicated that for acute DVT patient with iliac vein stenosis and good venous return, patients underwent thrombectomy combined with stenting had better efficacy compared with patients underwent thrombectomy alone with 6 months, while there was no significant difference in the symptom, signs, and quality of life between two groups after 6 months within a 2-year follow-up. This study provided meaningful guidance for the use of stents in this clinical setting. It was expected that through our and future research, more detailed intervention strategies for young DVT patients with iliac vein stenosis could be developed.

### Electronic supplementary material

Below is the link to the electronic supplementary material.

Supplementary Material 1

## Data Availability

Data are now available from the corresponding author (Xinwu Lu) on reasonable request.

## Abbreviations

ART: AngioJet rheolytic thrombectomy
CDT: Catheter-directed thrombolysis
DSA: Digital subtraction angiography
DVT: Deep-venous thrombosis
PTS: Postthromobtic syndrome
